# Circulating concentration of markers of angiogenic activity in patients with sarcoidosis and idiopathic pulmonary fibrosis

**DOI:** 10.1186/s12890-015-0110-3

**Published:** 2015-10-05

**Authors:** Dariusz Ziora, Dariusz Jastrzębski, Mariusz Adamek, Zenon Czuba, Jerzy Kozielski J., Alicja Grzanka, Alicja Kasperska-Zajac

**Affiliations:** Department of Lung Diseases and Tuberculosis, SMDZ in Zabrze, Medical University of Silesia, Katowice, Poland; Department of Thoracic Surgery, SMDZ in Zabrze, Medical University of Silesia, Katowice, Poland; Department of Immunology and Microbiology, SMDZ in Zabrze, Medical University of Silesia, Katowice, Poland; Department of Internal Diseases, Dermatology and Allergology, SMDZ in Zabrze, Medical University of Silesia, Katowice, Poland

## Abstract

**Background:**

Angiogenesis is an important process involved in the pathogenesis of diffuse parenchymal lung diseases. The aim of the study was to compare the angiogenic profile of patients with sarcoidosis and idiopathic pulmonary fibrosis (IPF) based on analysis of circulating factors.

**Methods:**

Serum concentrations of angiopoietin-2 (Ang-2), follistatin, granulocyte-macrophage-colony stimulating factor (GM-CSF), interleukin-8 (IL-8), platelet derived growth factor-BB (PDGF-BB), platelet endothelial cellular adhesion molecule-1 (PECAM-1) and vascular endothelial growth factors (VEGF) were measured in the patients and the healthy subjects.

**Results:**

Serum concentrations of G-CSF, follistatin, PECAM-1 and IL-8 were significantly higher in the IPF patients in comparison with the control group and the sarcoid patients. PDGF-BB concentrations were also significantly higher in serum of IPF patients than in sarcoid patients, but not than in the controls. In contrast, Ang-2 and VEGF concentrations did not differ significantly between the three groups. In the sarcoid patients, irrespective of the disease activity or the radiological stage, serum concentrations of these cytokines were similar to the control group.

**Conclusions:**

These results indicate that differences may exist in angiogenic activity between patients with parenchymal lung diseases. In contrast to sarcoidosis, IPF is characterized by a higher serum concentration of different molecules involved in the angiogenic processes .

## Background

The interstitial lung diseases are a heterogeneous group of diffuse parenchymal lung diseases, including sarcoidosis and idiopathic pulmonary fibrosis (IPF). IPF is characterized by tissue damage and exuberant repair with an aberrant wound-healing response leading to a severe disruption of the pulmonary architecture [1]. On the other hand, sarcoidosis is a multisystem inflammatory disease of unknown etiology that is characterized by non-caseating epithelioid cell granulomas and the accumulation of CD4-T cells and macrophages at the sites of inflammation [2, 3].

Angiogenesis, defined as the process of growth of new blood vessels, plays a pivotal role in wound healing and may contribute to the fibroproliferation and extracellular matrix deposition observed in IPF and in advanced stages of sarcoidosis [2–5].

Among various markers of angiogenesis, angiopoietin-2 (Ang-2), follistatin, granulocyte-macrophage-colony stimulating factor (GM-CSF), interleukin 8 (IL-8), platelet derived growth factor-BB (PDGF-BB), platelet endothelial cellular adhesion molecule-1 (PECAM-1) and vascular endothelial growth factor (VEGF), seem to be involved in different steps of physiological and pathological angiogenic processes, such as proliferation, maturation and survival of new blood vessels .

Ang-2, which is part of a family of vascular growth factors that play a role in embryonic and postnatal angiogenesis and is involved in controlling microvascular permeability, vasodilation, and vasoconstriction by signaling the smooth muscle cells surrounding vessels [6, 7]. In addition, Ang-2 as an antagonist ligand of Tie2 receptor expressed by endothelial and hematopoietic cells, promotes cell death and disrupts vascularization [6, 7]. Follistatin is an activin-binding protein with the function of binding and bioneutralizing members of the transforming growth factor beta (TGF-β) superfamily, with a particular focus on activin, a paracrine hormone [8]. IL-8, known as a neutrophil chemotactic factor and produced by macrophages, epithelial cells, airway smooth muscles and endothelial cells, is also a potent promoter of angiogenesis [9].

GM-CSF is a cytokine secreted by macrophages, T cells, mast cells, NK cells, endothelial cells and fibroblasts and it functions as a white blood cell growth factor. Additionally, GM-CSF plays a vital role in angiogenesis through the regulation of VEGF and sVEGFR-1 [10]. PDGF-BB stimulates proliferation of fibroblasts [11]. PECAM- 1 plays role in the leukocyte transmigration [12]. VEGF, which is an endothelial cell-specific mitogen that promotes angiogenesis and is a potent mediator of vascular permeability [13, 14].

The aim of this study was to analysis serum concentrations of these markers associated with angiogenic processes, rarely investigated simultaneously in patients with IPF and sarcoidosis; looking for any possible differences and correlations with the main clinical parameters.

## Methods

Forty three non-smoking patients with sarcoidosis and 17 non-smoking patients with IPF characterized in our previous report [15] were enrolled in the study. Their demografic and spirometric characteristics are shown in Table 1. The recruitment of patients with blood serum collection was completed in 14 months – February 2012 till March 2013. Sarcoidosis was histologically proven according to the ATS criteria [2] (i.e., by the demonstration of non-caseating granulomas in tissue samples obtained in the transbronchial lung biopsy, the lymph node scale biopsy, mediastinoscopy, or the bronchial biopsy). Only patients with clinical stages I - III were included in the study. Sarcoidosis stage I is featured by hilar lymph nodes enlargement, stage II by concomitant hilar lymphadenopathy and pulmonary infiltrates, whereas in stage III only pulmonary infiltrates are visible on the chest X-ray. Eleven patients had stage I, 20 patients had stage II, and 12 patients had stage III of the disease [2]. Among the patients with sarcoidosis, 18 subjects had a progressive disease, i.e., progressive lymphadenopathy or interstitial infiltrates with or without deterioration of the pulmonary function tests (at least FVC or FEV1 decline above 10 % during 3 month period before study enrollment) [15]. None of the patients with sarcoidosis had Löffgren syndrome.

**Table 1:** Demografic and spirometric data patients with IPF and sarcoidosis

	Sarcoidosis total	IPF	Controls
	*N* = 43 (20 F/ 23 M)	*N* = 17 (7 F/ 10 M)	*N* = 20 (10 F/10 M)
	(mean ± SEM)	(mean ± SEM)	(mean ± SEM)
	Median (Interquartile range)	Median (Interquartile range)	Median (Interquartile range)
Age (years)			
	35.7 ± 1.3	58.3 ± 1.6 ***	34.3 ± 1.2
	33.5 (29.5–40.0)	58.0 (53.7–64.0)	33.5 (29.5–37.5)
BMI (kg/m^2^)	26.9 ± 0.5	27.4 ± 0.7	27.2 ± 0.6
	27.0 (23.2–31.4)	27.6 (21.2–31.3)	27.1 (22.3–30.1)
FEV1 (% of predicted)			
	86.1 ± 1.6	67.9 ± 2.3 **	
	85.5 (81.0–93.0)	69.0 (61.0–77.3)	
FVC (% of predicted)			
	96.2 ± 2.1	70.3 ± 1.8 **	
	98.0 (88.5–104.0)	69.0 (63.5–79.0)	
FEV1%VC (%)			
	75.7 1.0	77.0 ± 1.8	
	75.0 (71.0–80.0)	76 (73.5–82.3)	

*F* females, *M* males, *IPF* idiopathic pulmonary fibrosis, *FEV1* Forced expiratory volume in 1 s

*FVC* Forced Vital Capacity, *FEV1%VC* forced expiratory volume in one second % of vital capacity **p < 0.001 IPF vs sarcoidosis ***p < 0.001 IPF vs controls and sarcoidosis

Seventeen patients with IPF were diagnosed according to the ATS criteria [1] (using the HRCT and/or lung biopsy). The mean duration of the disease since the diagnosis was 3.5 years (0.5–6 years) for sarcoidosis and 1.2 year (0.5–3 years) for IPF. No study participant was taking oral corticosteroids or immunosuppressive drugs at the time of the enrollment to the study. None of the patients or controls suffered from any chronic liver or kidney disease; all participants were free from infections for at least three months before the blood sample collection. All patients were candidates for Nordic walking rehabilitation and were routinely controlled in the out-patient clinic at the Department of Lung Diseases and Tuberculosis, Silesian University of Medicine, and underwent radiological and spirometric examinations. Spirometry was performed using the PULM-Test 1000 spirometer (Krakow, Poland) according to the ATS/ERS criteria [16], and the results were expressed as the percentage of the predicted values [17]. Twenty healthy nonsmoking volunteers matching the sarcoid group in terms of sex, age, and BMI served as controls.

All subjects provided written informed consent and the study was approved by the local ethical committee at Medical University of Silesia (number KNW/0022/KB1/32a/12).

### Analytical methods

Serum concentrations of Ang-2,follistatin, GM-CSF, IL-8, PDGF-BB, PECAM-1 and VEGF were measured in duplicate (the mean value from 2 measurements was derived) using commercially available multiplex bead-based sandwich immunoassay kits (Human Angiogenesis 9-Plex Panel, Bio-Rad Laboratories). All assays were performed according to the manufacturer’s instructions. The system allowed simultaneous identification of 9 different molecules in a 96-well filter plate. We used 9 different sets of fluorescent dyed beads with capture monoclonal antibodies specific for each molecule to be analyzed. The appropriate molecule standards (50 μl/well) and samples diluted (50 μl/well) in plasma diluents were added to a 96-well filter plate and incubated for 30 min. at room temperature. After 3 washes, premixed streptavidin/phycoerythrin was added to each well and incubated for 10 min. followed by 3 additional washes. The beads were resuspended with 125 μl of assay buffer, and the molecule reaction mixture was quantified via the Bio-Plex protein array reader.

The data analysis was performed using the Bio-Plex Manager software version 4.1.1. (Bio-Rad Laboratories). Values with a coefficient of variation over 12 % were discarded before the final data analysis. The concentration (pg/mL) of different analytes in the plasma samples was identified using standard curves from the multiplex assays. The minimum level of detection for VEGF was 3.27, 11.32 for Ang-2, 5.15 for PDGF-BB, 33.29 for PECAM-1, 8.41 for follistatin, 6.66 for G-CSF and 1.6 for IL-8.

### Statistical analysis

The statistical analysis was conducted using Statistica 6.0 software (StatSoft Inc., Tulsa, OK). The normal data distribution was assessed using Shapiro-Wilk test. Homogeneity of variance was evaluated using Levene’s test. Comparisons between the study groups were performed using the ANOVA, Mann–Whitney or Kruskal-Wallis test, if the distribution of data was abnormal. Correlations were analyzed by Spearman’s test. All results were statistically significant if *p* < 0.05.

## Results

**Fig. 1:**
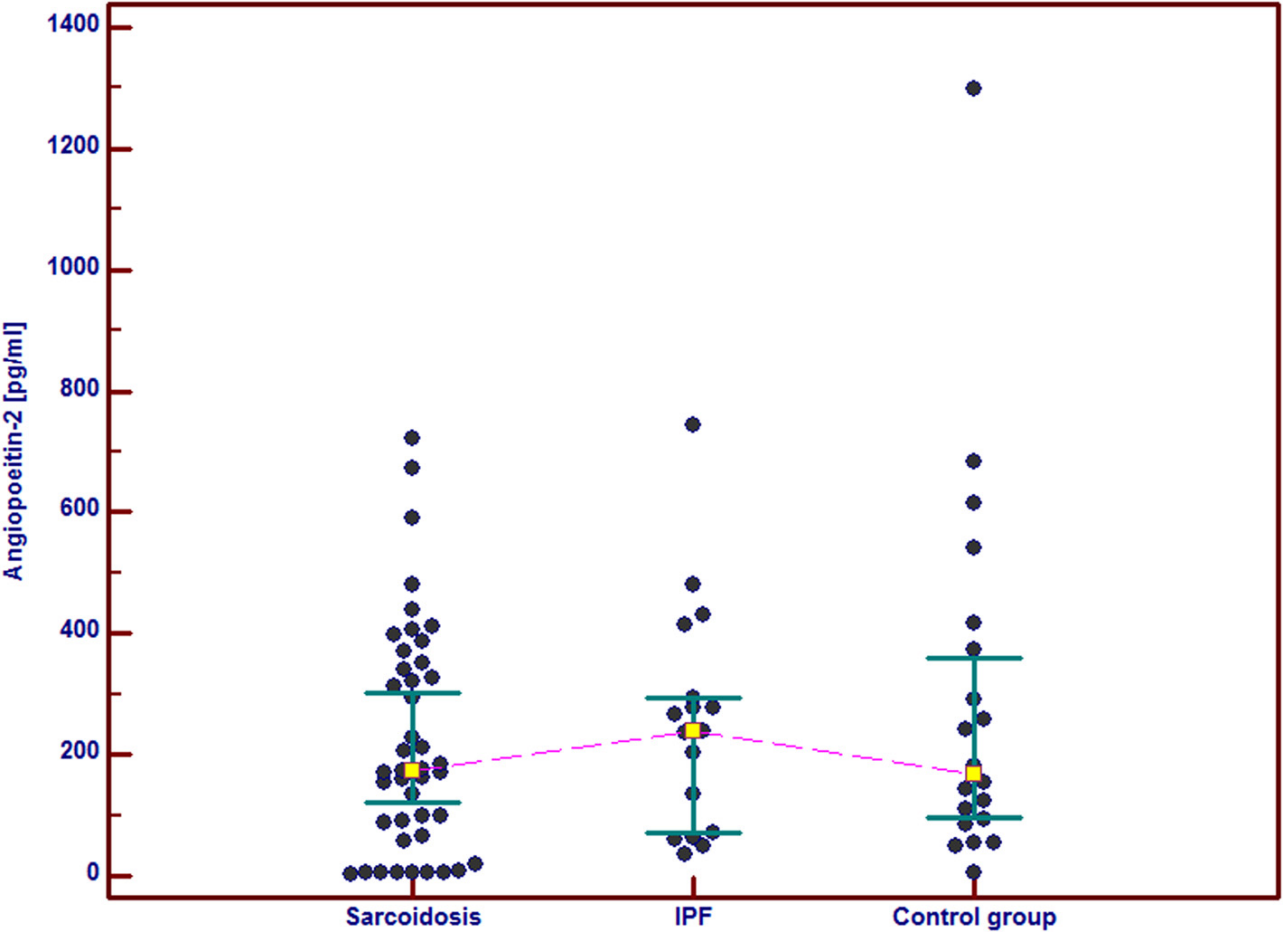
Individual serum concentrations of angiopoeitin-2 in patients with sarcoidosis and IPF in comparison to the control group. IPF vs sarcoidosis and vs control group, *p* > 0.05

**Fig. 2:**
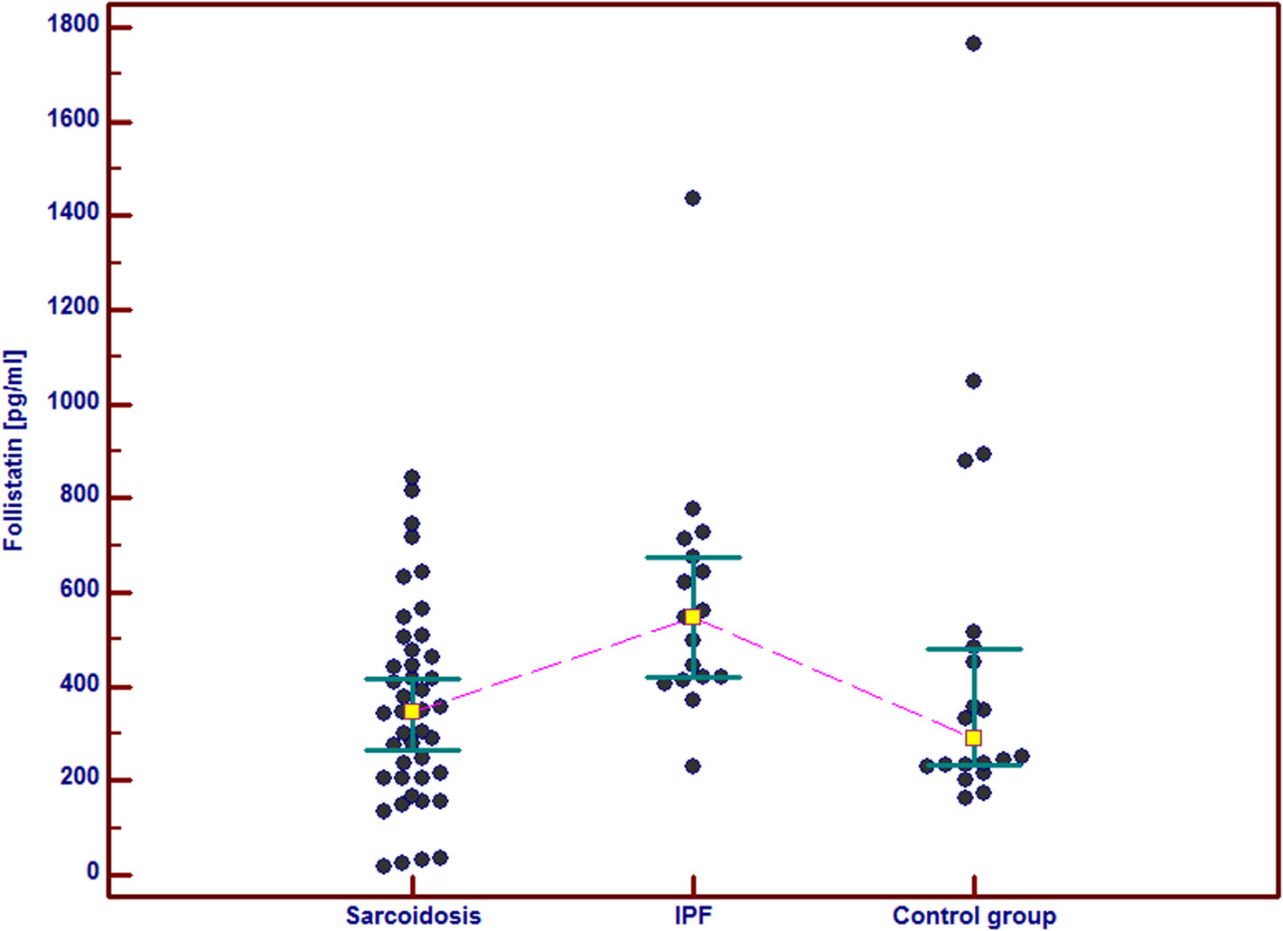
Individual serum concentrations of follistatin in patients with sarcoidosis and IPF in comparison to the control group. IPF vs sarcoidosis and vs control group, *p* < 0.01

**Fig. 3:**
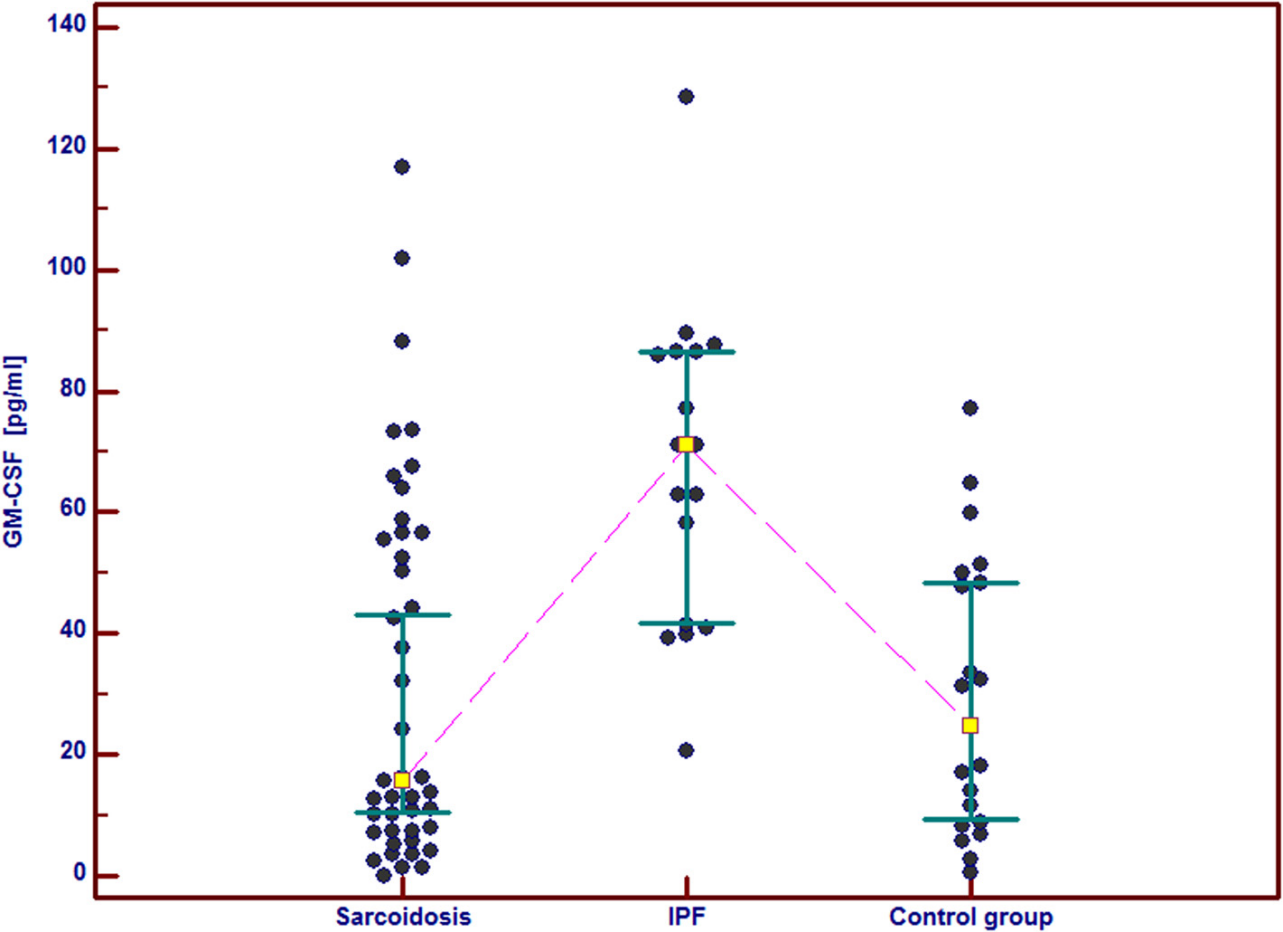
Individual serum concentrations of GM-CSF in patients with sarcoidosis and IPF in comparison to the control group. IPF vs sarcoidosis and vs control group, *p* < 0.001

**Fig. 4:**
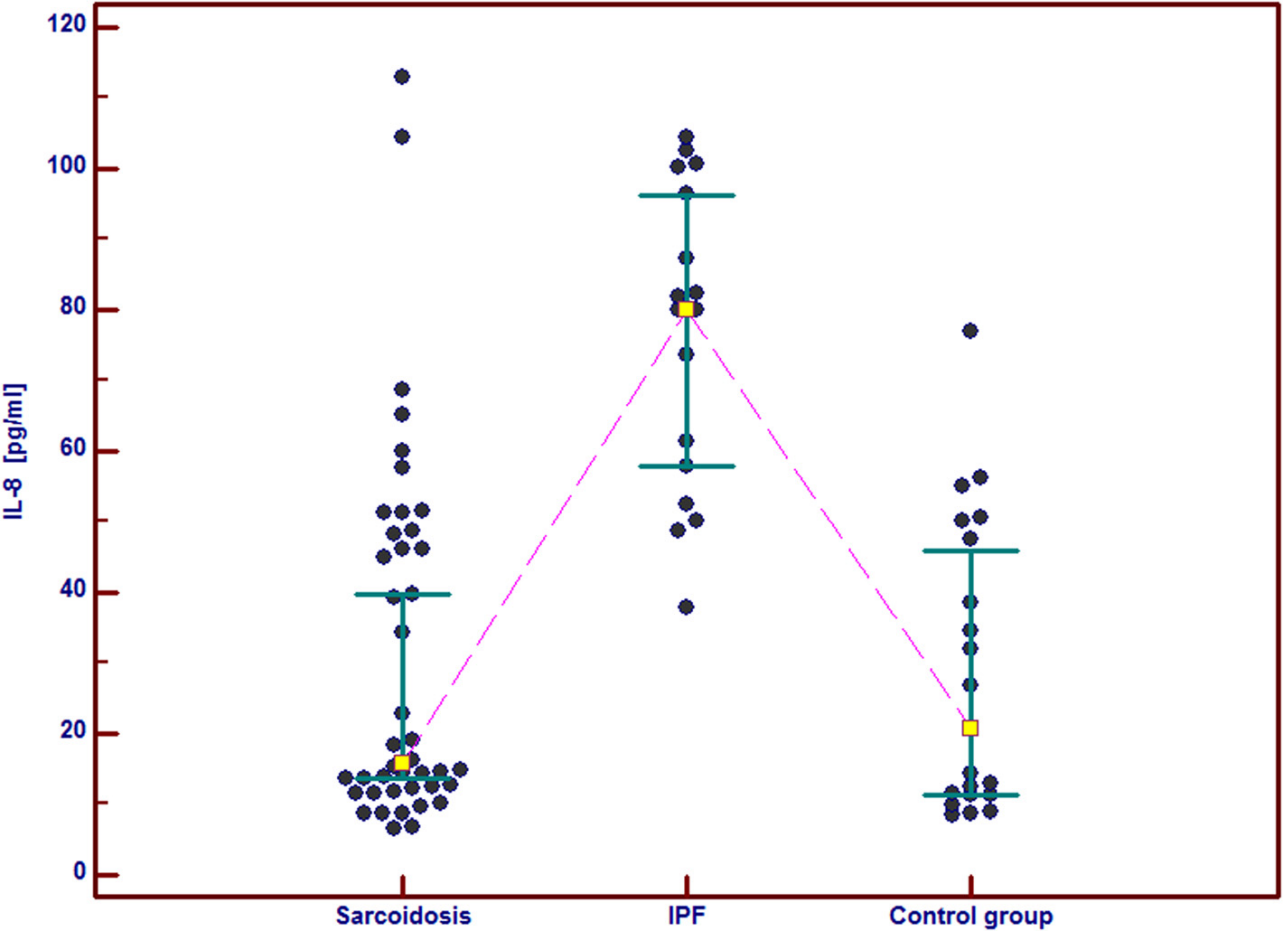
Individual serum concentrations of IL-8 in patients with sarcoidosis and IPF in comparison to the control group. IPF vs sarcoidosis and vs control group, *p* < 0.001

**Fig. 5:**
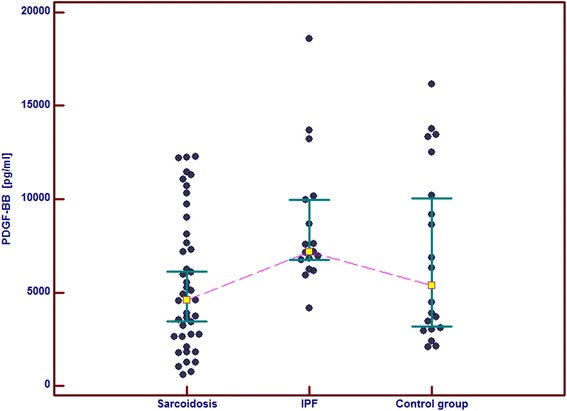
Individual serum concentrations of PDGF-BB in patients with sarcoidosis and IPF in comparison to the control group. IPF vs sarcoidosis, *p* < 0.05

**Fig. 6:**
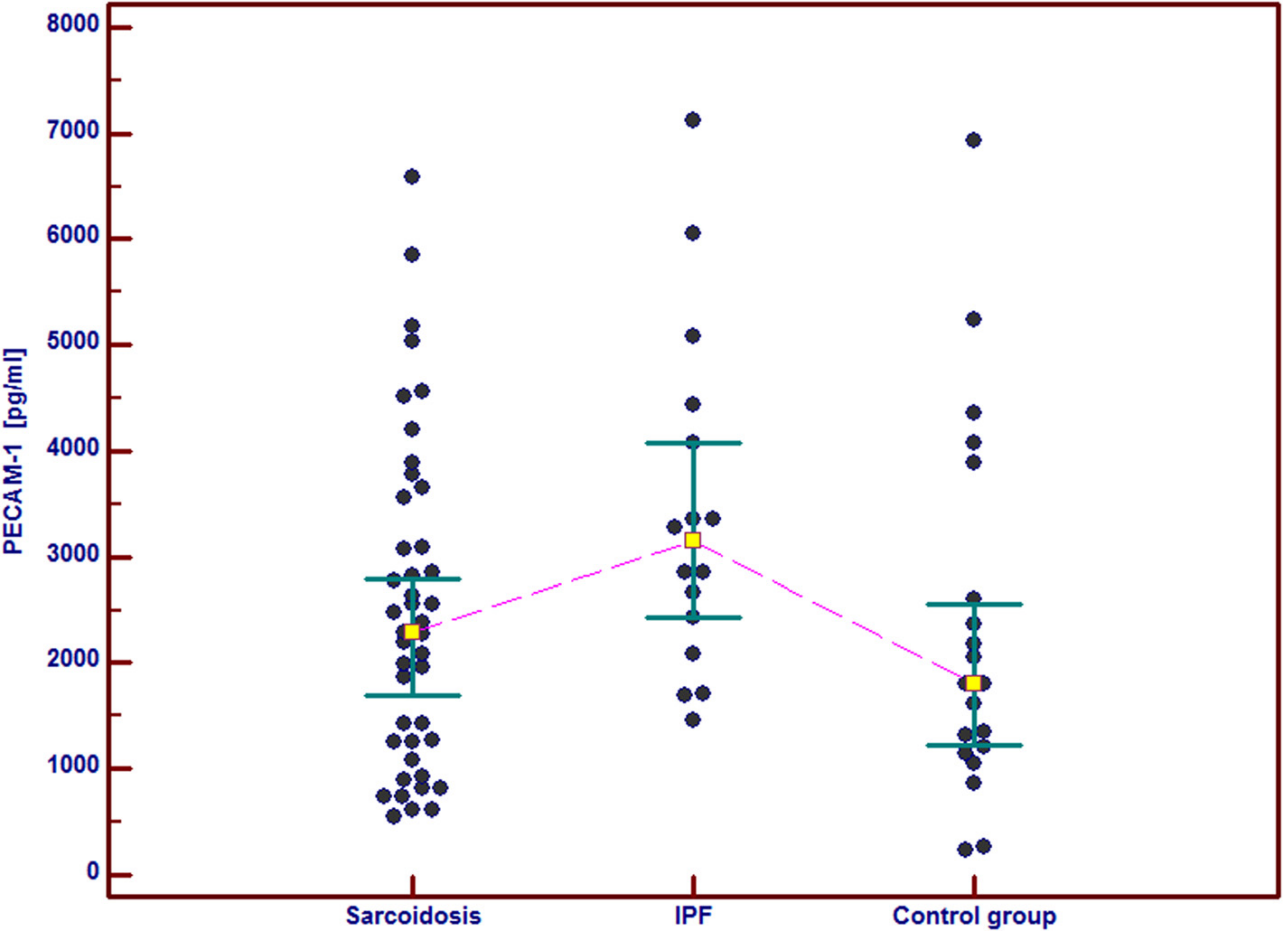
Individual serum concentrations of PECAM-1 in patients with sarcoidosis and IPF in comparison to the control group. IPF vs sarcoidosis and vs control group, *p* < 0.05

**Fig. 7:**
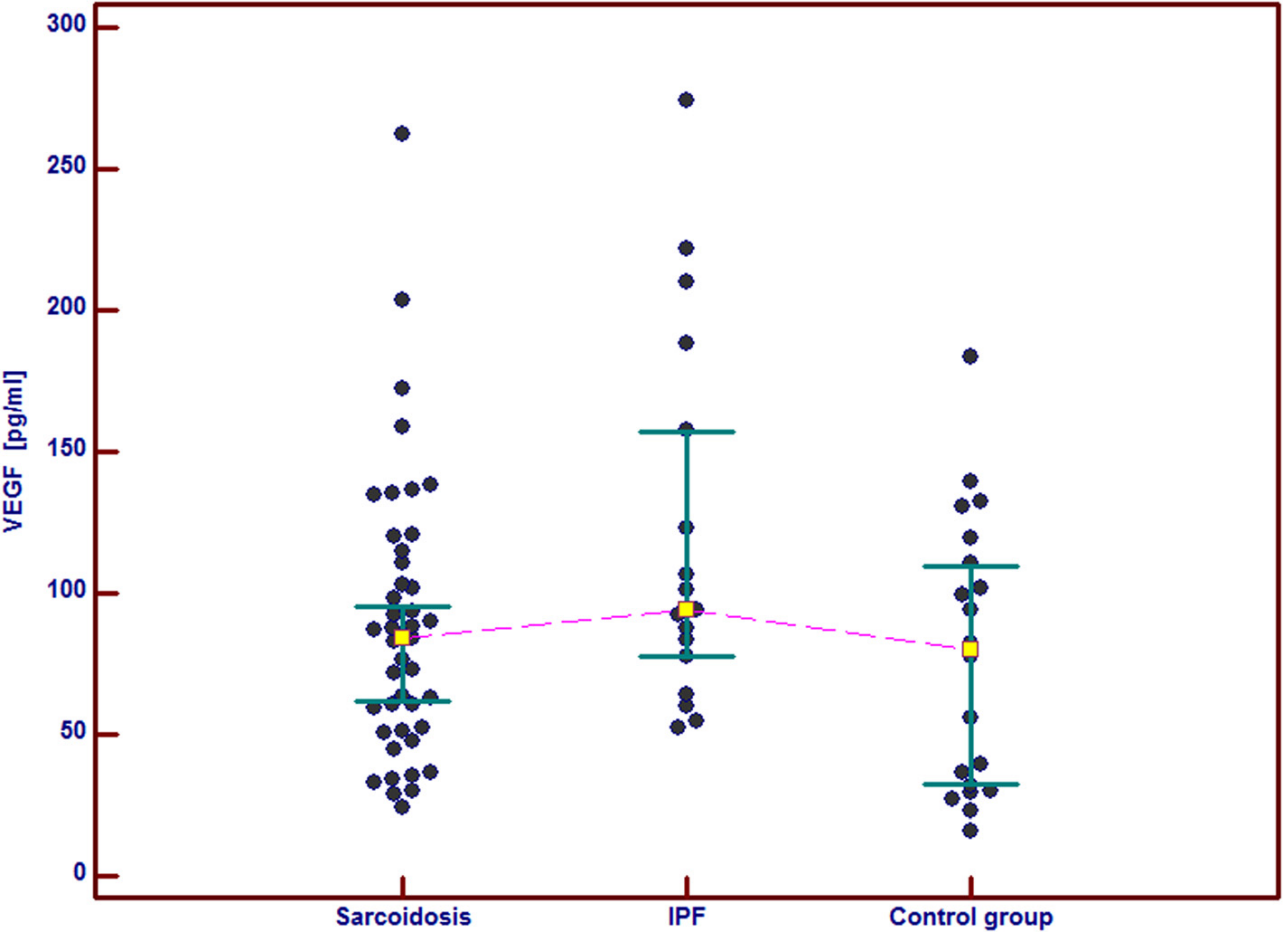
Individual serum concentrations of VEGF in patients with sarcoidosis and IPF in comparison to the control group. IPF vs sarcoidosis and vs control group, *p* > 0.05

In the IPF patients in comparison with the control group and the sarcoid patients we observed significantly higher serum concentrations of GM-CSF, follistatin, PECAM-1 and IL-8. Serum concentrations of PDGF-BB were also significantly higher in the IPF patients than in the sarcoid patients but not than in the controls. In the IPF patients, serum Ang-2 and VEGF concentrations did not differ significantly from these obtained in the sarcoid group and the controls (Table 2 and Figs. 1, 2, 3, 4, 5, 6, and 7). In the IPF group we found statistically significant correlations between concentrations of Ang-2 and IL-8 (*r* = 0.62, *P* < 0.01), follistatin and PECAM-1 (*r* = 0.83, *P* < 0.0001) and PDGF-BB and VEGF (*r* = 0.7, *P* < 0.01).

**Table 2:** The mean serum concentrations of examined cytokines in patients with IPF and sarcoidosis

	Sarcoidosis *n* = 43	IPF *n* = 17	Control group *n* = 20
	Mean ± SEM	Mean ± SEM	Mean ± SEM
	Median (95 % CI)	Median (95 % CI)	Median (95 % CI)
Angiopoeitin-2	221.5 ± 28.7	251.6 ± 45.6	288.8 ± 68,9762
(pg/ml)	173.5 (120.7–301.8)	237.8 (72.1–294.6)	168.7 (95.6–360.4)
Follistatin	357.6 ± 31.9	582.5 ± 64.5**	462.7 ± 89.6
(pg/ml)	346.5 (264.7–416.1)	545.8 (418.5–674.5)	291.2 (231.5–478.3)
GM-CSF	31.7 ± 4.7	67.6 ± 6.4***	29.5 ± 5.2
(pg/ml)	15.7 (10.5–43.1)	71.1 (41.7–86.5)	24.8 (9.3–48.2)
IL-8	30.8 ± 4.0	76.3 ± 5.2***	28.9 ± 4.7
(pg/ml)	15.8 (13.8–39.7)	79.9 (57.9–96.3)	20.7 (11.4–46.0)
PDGF-BB	5528.3 ± 551.2	8629.8 ± 869.1^#^	7080.1 ± 1046.6
(pg/ml)	4607.0 (3464.6–6128.8)	7178.0 (6732.8–9948.4)	5384.1 (3173.5–10040.3)
PECAM-1	2490.4 ± 232.9	3392.1 ± 376.8*	2316.9 ± 388.9
(pg/ml)	2290.7 (1693.7–2795.2)	3147.5 (2428.6–4072.6)	1798.1 (1226.1–2563,1)
VEGF	88.7 ± 7.7	120.5 ± 16.0	78.0 ± 10.8
(pg/ml)	84.1 (61.9–95.4)	94.3 (77.6–157.2)	80.1 (32.6–109.2)

*p < 0.05 IPF vs sarcoidosis and vs control group

**p < 0.01 IPF vs sarcoidosis and vs control group

***p < 0.001 IPF vs sarcoidosis and vs control group

^#^ *p* < 0.05 IPF vs sarcoidosis

In the sarcoid patients concentrations of serum Ang-2, follistatin, GM-CSF, IL-8, PDGF-BB, PECAM-1 and VEGF were similar to the values noticed in the control group. In patients with progressive and non-active (clinically stable) sarcoidosis the serum concentrations of all molecules were similar. No significant differences were observed between stage I, II and III-only serum Ang-2 concentrations in stage I patients was significantly higher than in stage II and III (Table 3). In the sarcoidosis group we found statistically significant relationships between concentrations of Ang-2 and IL-8 (*r* = 0.62, *P* < 0.0001), follistatin and PECAM-1 (*r* = 0.76, *p* < 0.0001) as well as PDGF-BB and PECAM-1 (*r* = 0.82, *p* < 0.0001).

**Table 3:** The mean spirometric values and the mean serum concentrations of examined cytokines in patients with progressive and non-active sarcoidosis and in radiological stage I,II and III

	Progressive, active sarcoidosis *n* = 18	Non active sarcoidosis *n* = 25	Sarcoidosis Stage I *n* = 10	Sarcoidosis Stage II *n* = 20	Sarcoidosis Stage III *n* = 13
	(mean ± SEM)	(mean ± SEM)	(mean ± SEM)	(mean ± SEM)	(mean ± SEM)
	Median (Interquartile range)	Median (Interquartile range)	Median (Interquartile range)	Median (Interquartile range)	Median (Interquartile range)
Age (years)	35.1 ± 1.9	36.2 ± 1.7	30.3 ± 1.5	35.7 ± 1.9	40.3 ± 2.3
	32.5 (30.0–40.0)	34.0 (29.0–40.0)	29.0 (29.0–30.8)	33.5 (30.5–39.0)	40.0 (33.5–44.3)
FEV1 (% of predicted)	87.1 ± 2.0	85.4 ± 2.4	87.7 ± 2.7	87.2 ± 1.8	83.1 ± 4.3
	84.0 (81.0–92.0)	87.0 (80.0–94.0	90.0 (82.8–101.0)	85.0 (80.5–97.3)	85.0 (81.0–91.5)
FVC (% of predicted)	96.8 ± 3.4	95.9 ± 2.7	100.2 ± 3.7	97.6 ± 2.8	90.7 ± 4.6
	95.0 (88.0–108.0)	98.0 (91.0–103.0)	100.0 (96.0–105.5)	100.0 (89.5–104.0)	90.0 (82.8–101.0)
FEV1%VC (%)	75.6 ± 1.8	75.7 ± 1.2	77.0 ± 1.8	74.8 ± 1.5	75.8 ± 2.2
	74.5 (70.0–82.0)	75.5 (71.0–79.0)	77.0 (74.0–80.0)	74.0 (70.5–77.5)	75.0 (68.8–83.5)
Angiopoeitin-2 (pg/ml)	239.1 ± 37.9	208.4 ± 41.5	355.6 ± 66.0*	206.0 ± 37.9	142.2 ± 42.2
	220.0 (120.1–380.6)	169.0 (69.9–281.5)	361.7 (170.0–518.8)	178.3 (98.6–310.0)	89.0 (13.5–192.9)
Folistatin (pg/ml)	372.9 ± 51.9	346,456 ± 41.1	448.5 ± 59.1	328.8 ± 46.4	331.9 ± 62.2
	348.8 (258.6–466.7)	304.6 (206.1–444.0)	406.5 (265.1–636.5)	296.6 (211.7–416.8)	357.9 (154.5–472.3)
GM-CSF (pg/ml)	28.8 ± 8.0	33.7 ± 5.7	45.4 ± 12.9	32.4 ± 6.0	20.1 ± 6.9
	12.8 (10.3–32.0)	32.1 (7.6–56.0)	33.4 (10.5–88.2)	24.2 (8.3–52.1)	12.7 (2.5–34.6)
IL-8 (pg/ml)	28.4 ± 7.7	32.4 ± 4.4	47.7 ± 11.8	29.0 ± 4.6	20.4 ± 4.7
	14.5 (13.7–22.7)	39.2 (11.9–48.7)	42.8 (15.1–85.7)	18.4 (13.3–46.6)	12.8 (10.7–28.8)
PDGF-BB (pg/ml)	5304.0 ± 786.5	5689.8 ± 772.3	5865.1 ± 856.4	5564.5 ± 863.6	5213.5 ± 1123.2
	4591.3 (3054.5–6419.3)	5547.9 (2724.6–7594.7)	5044.5 (3544.5–8742.6)	4757.8 (2655.4–7972.2)	4575.5 (1536.6–8612.5)
PECAM-1 (pg/ml)	2537.3 ± 330.4	2456.6 ± 327.9	3126.1 ± 328.6	2377.3 ± 394.0	2175.5 ± 389.7
	2469.8 (1502.1–3418.4)	2190.3 (1295.8–2854.1)	2596.1 (2323.9–4164.1)	1926.5 (1262.9–2818.9)	2290.7 (862.3–3340.9)
VEGF (pg/ml)	93.9 ± 13.7	84.9 ± 8.6	116.8 ± 21.5	87.9 ± 9.4	68.29 ± 9.6
	79.9 (60.6–117.8)	87.6 (53.5–101.2)	98.8 (61.5–165.9)	87.9 (60.4–100.3)	62.7 (34.3–100.4)

*p < 0.05 sarcoidosis stage I vs sarcoidosis stage II and III

Both in the IPF group and the sarcoid group no significant correlations between concentrations of the examined molecules and the pulmonary function parameters (FEV1, FVC) (Table 3).

## Discussion

In our study elevated serum concentrations of IL-8, GM-CSF, follistatin, PDGF-BB and PECAM-1 in the IPF patients, but not in the sarcoidosis patients even in active progressive disease have been observed. Surprisingly, VEGF and Ang-2 concentrations (i.e., the main proangiogenic cytokine) both in the sarcoid and the IPF patients were similar to the concentrations observed in the controls. We did not find any differences in serum concentrations of the examined cytokines obtained from patients with progressive i.e., active sarcoidosis and stable non-active sarcoidosis. To the best of our knowledge we were the first to demonstrate the serum concentrations of Ang-2, PECAM-1 and follistatin in IPF and sarcoidosis.

Keane et al. [18, 19] demonstrated increased angiogenic activity in a large number of IPF lung specimens and speculated that there may be an opposing balance of angiogenic and angiostatic factors favors angiogenesis. However, other reports made the role of angiogenesis in IPF controversial. Meyer et al. [20] and Koyama et al. [21] documented depressed VEGF levels in bronchoalveolar lavage (BAL) from IPF patients compared to a variety of diffuse parenchymal lung diseases or healthy controls. On the other hand, a statistically significant increase has been detected in VEGF mRNA expression in BAL from IPF in comparison with pulmonary sarcoidosis. However, no statistically significant difference has been measured in VEGF concentration between patients with IPF and sarcoidosis [22].

Nevertheless, Sekiya et al. [23] demonstrated a strong correlation between elevated serum VEGF concentrations and clinical parameters of the disease activity and severity in patients with sarcoidosis, indicating their potential usefulness as a predictor of the disease extent and the responsiveness to treatment. Our previous study [24] revealed increased VEGF concentration in the BAL fluid obtained from the lung regions most affected by the sarcoid process. Tzouvelekis et al. [25], using the tissue microarray technology, demonstrated an abundant expression of VEGF in sarcoidosis mediastinal lymph nodes. An elevated serum concentration of IL-8 in IPF can indicate the disease activity [26]. IL-8 was increased and correlated with the percentage and absolute number of neutrophils in the BAL fluid of IPF patients [27].

In patients with sarcoidosis we previously observed significantly higher concentrations of GM-CSF in the BAL fluid from patients with active sarcoidosis in comparison with the control group [28]. Recently, Patterson et al. [29] have demonstrated a trend towards a significant increase in GM-CSF serum concentrations in non-fibrotic pulmonary sacroidosis, but not in fibrotic pulmonary sarcoidosis. In present study, GM-CSF serum concentration were significantly higher in IPF, but not in sarcoidosis.

We noticed significant elevation of PDGF in serum of IPF patients. Cao et al. [30] demonstrated that PDGF-BB was located in pulmonary macrophages, fibroblasts and vascular smooth muscle and endothelial cells, which suggests the association of PDGF with fibroplasia and the deposition of extracellular matrix, as well as vessel remodeling in IPF. Our study stays in line with Wolff et al. [31], who demonstrated significantly elevated PDGF-BB levels in the BAL fluid from patients with allergic alveolitis and fibrosing alveolitis, but decreased in patients with sarcoidosis.

We observed an increased serum concentration of follistatin in IPF, but not in sarcoidosis. Follistatin is effective in treating bleomycin -induced fibrosis by blocking the actions of activin and TGF-beta [32]. Recently Myllärniemi et al. found high-intensity follistatin staining in the activated alveolar epithelium and parenchymal macrophages obtained from IPF patients and suggest that activin-B and follistatin may be useful as biomarkers of IPF [33].

In our study serum concentrations of ANG-2 were also similar in IPF, sarcoidosis and the controls. Serum Ang-2 concentrations in stage I was higher than in stage II and III patients with saroidosis. ANG-2 is an essential factor for the vascular formation and is normally expressed in the endothelial remodeling tissues, while it is higher expressed during hypoxic processes [6]. Recently a significant reduction of Ang-1 and increase of Ang-2 protein expression in BAL fluid were detected in IPF [34].

The low angiogenetic serum activity in our patients with sarcoidosis may be, at least in part, explained by the findings of Kambouchner et al. [35]. These authors have not observed any angiogenesis in pulmonary sarcoidosis. Namely CD31 immunolabelling, which recognizes PECAM-1 on the blood micro-vessel network, revealed that 85 % of intralobular sarcoid granulomas were very poorly supplied by blood capillaries at the outer edge of the peripheral fibrous ring, far from the cellular compartment [35]. However, granulomas were closely associated with lymphatics. This finding suggests that a large majority of pulmonary sarcoid granulomas can be considered ‘avascular’ [35].

We are not able to replicate some previously published results of circulating cytokine alterations. Methodological differences may account for our different, yet not contradictory findings: whereas many studies have employed single-cytokine ELISA tests, used plasma or culture supernatant samples, or had cohorts of newly diagnosed disease, we employed a multiplex assay, used serum samples, and had a cohort of chronic disease.

Our study has limitations. A relatively small number of patients, especially with IPF, was included into the study and cytokines examinations were performed only in serum, but not in BAL. The results of Diffusing Capacity of the Lungs for Carbon Monoxide (DLCO) were available only in few patients as no correlations were estimated between DLCO and cytokines concentrations. No relationships were checked between the examined angiogenic molecules and other potential markers of sarcoidosis or IPF activity, for example: ACE (angiotensin converting enzyme) or LDH (lactate dehydrogenase), as no such data were available. It would be interesting to compare results obtained from patients with IPF with those at stage IV of sarcoidosis i.e., with fibrotic lung changes, it was very difficult however to collect a sufficient number of previously untreated sarcoid patients in IV stage.

## Conclusions

These results indicate that differences may exist in angiogenic activity between patients with parenchymal lung diseases. In contrast to sarcoidosis, IPF is characterized by a higher serum concentration of different molecules involved in the angiogenic processes.

## Abbreviations

IPF: Idiopathic pulmonary fibrosis
Ang-2: Angiopoietin-2
GM-CSF: Granulocyte-macrophage-colony stimulating factor
IL: Interleukin
PDGF-BB: Platelet derived growth factor-BB
PECAM-1: Platelet endothelial cellular adhesion molecule-1
VEGF: Vascular endothelial growth factors
TGF-β: Transforming growth factor beta
FVC: Forced vital capacity
FEV1: Forced expiratory volume in 1 s
BMI: Body mass index
BAL: Bonchoalveolar lavage
DLCO: Diffusing capacity of the lungs for carbon,monoxide
ACE: Angiotensin converting enzyme
LDH: Lactate dehydrogenase
